# Rumen and plasma metabolomics profiling by UHPLC-QTOF/MS revealed metabolic alterations associated with a high-corn diet in beef steers

**DOI:** 10.1371/journal.pone.0208031

**Published:** 2018-11-28

**Authors:** You Yang, Guozhong Dong, Zhi Wang, Jian Wang, Zhu Zhang, Junhui Liu

**Affiliations:** College of Animal Science and Technology, Southwest University, Chongqing, China; University of Illinois, UNITED STATES

## Abstract

High-grain diets are strongly associated with metabolic disorders in beef steers. Metabolomics can be used to explore the relationship between diet and metabolic changes, but no study has reported rumen and plasma metabolomics profiling associated with increasing dietary corn proportions in the diet of beef steers. Therefore, 12 steers paired according to similar body weights and body condition scores were randomly allocated to one of two diets: a low-corn (28.76%) diet (LCD) with a 40:60 ratio of concentrate to roughage and a high-corn (48.76%) diet (HCD) with a 60:40 ratio of concentrate to roughage. Metabolomics profiling by ultra-high-performance liquid tandem chromatography quadrupole time of flight mass spectrometry (UHPLC-QTOF/MS) showed that steers fed the HCD had increased rumen and plasma carbohydrate metabolites and amino acids, which contributed to the growth of the beef steers. However, the rumen acidity and ruminal and plasma lipopolysaccharide (LPS) concentrations significantly increased with the increase amounts of corn in the diet. In total, 717 rumen metabolites and 386 plasma metabolites were identified. By multivariate analysis, 144 rumen and 56 plasma metabolites were further identified that were significantly different between the two groups (*P* < 0.05 and variable influence on projection > 1). The differential metabolites in the rumen and plasma were associated with different metabolic pathways, and phenylalanine, tyrosine and tryptophan biosynthesis and phenylalanine metabolism were common key metabolic pathways for the two biofluids. In conclusion, the high-corn diet improved the growth performance of beef steers but decreased the ruminal pH and increased the LPS and harmful metabolites in the rumen and blood, which has implications for the incidence of metabolic diseases. The identified differential metabolites in both the rumen and plasma and the related metabolic pathways may contribute to the exploration of potential biomarkers for high-corn diet-based metabolic diseases.

## Introduction

In beef production, steers are often fed high-grain diets to increase their growth rate or due to an intermittent forage shortage. Feeding beef cattle high-grain diets rich in starch results in increased yields of volatile fatty acids (VFAs) and lactic acid in the rumen [1]. Rumen pH values decrease once these acids accumulate in the rumen [2], and subacute ruminal acidosis (SARA) occurs if the rumen pH is less than 5.6 for more than 3 h/day [3]. During SARA, lipopolysaccharide (LPS) is released in a large amount in the rumen and large intestine [4]. The translocation of LPS into the circulation can lead to systemic inflammatory responses, inducing metabolic alterations and perturbation in blood metabolites [5].

Although some studies have described several events as associated with feeding high-grain diets, most studies have focused mainly on a single class of ruminal fluid metabolites, such as amino acids, carbohydrates or VFAs. Recent advances using high-throughput quantitative metabolomics methods have opened new avenues to address nutritional problems in ruminants [6, 7]. Moreover, plasma metabolites can also be used to monitor the metabolic health status of ruminants [8]. However, metabolomics information for beef cattle is missing [9–12], and most metabolic studies have mainly concentrated on a single biofluid, such as the rumen fluid of dairy cows [13].

Metabolomics is used in diet-challenge experiments to extract overall metabolic information and explore complicated metabolic pathways. Common metabolomics techniques include ^1^H-NMR (^1^H-nuclear magnetic resonance), GC-MS (gas chromatography-mass spectrometry) and LC-MS (liquid chromatography-mass spectrometry) [14]. Compared with ^1^H-NMR and GC-MS, LC-MS has been widely used in metabolomics studies due to its high resolution, detection sensitivity and nonderivatization of samples [15, 16]. Ultra-high-performance liquid tandem chromatography quadrupole time of flight mass spectrometry (UHPLC-QTOF/MS) was developed based on LC-MS and can determine and quantify more metabolites [15].

In this study, we used UHPLC-QTOF/MS to perform both rumen and plasma metabolomics profiling and to evaluate the relationship between a high-corn diet and metabolic alterations in beef steers.

## Materials and methods

### Ethics statement

The experiment was approved by the Animal Ethics Committee of Southwest University. The experimental procedures, including animal care, complied strictly with the ‘‘Guidelines on Ethical Treatment of Experimental Animals (2006, No. 398)” issued by the Ministry of Science and Technology of China and the “Regulations on the Management of Experimental Animals (2006, No. 195)” issued by Chongqing Municipal People’s Government.

### Experimental design

Twelve healthy Charolais × Luxi hybrid steers on the experimental farm of the university were selected with an initial body weight (BW) of 365.34 ± 42.96 kg and similar body condition scores of 3.20 ± 0.56 for this study. And there was no significant difference in the rumen and plasma baseline metabolomics profiling among the animals (S1 Fig). Using a paired design, the steers were paired according to similar BWs and body condition scores and randomly allocated to one of two diets (Table 1).

**Table 1:** Ingredients and nutrient compositions of the experimental diets (dry matter basis).

Ingredients (%)	Treatment^a^	
	LCD	HCD
**Chinese wild rye**	31.50	11.50
**Sorghum distiller’s grains**	28.50	28.50
**Corn grain**	28.76	48.76
**Rapeseed meal**	8.61	8.61
**Salt**	0.30	0.30
**Limestone**	2.05	2.05
**Premix**^**b**^	0.28	0.28
**Nutrients (%)**		
**Crude protein**	12.9	12.6
**Ether extract**	3.8	3.1
**Neutral detergent fiber**	43.1	35.6
**Acid detergent fiber**	21.7	13.1
**Crude ash**	5.0	4.6
**Calcium**	0.33	0.35
**Total phosphorus**	0.27	0.28

^a^LCD (the low-corn diet), the concentrate to roughage ratio was 40:60; HCD (the high-corn diet), the concentrate to roughage ratio was 60:40, and 20% of the Chinese wild rye was replaced with 20% corn to constitute a high-corn diet.

^b^Formulated to provide (per kg diet) Fe (as ferrous sulfate) 50 mg, Cu (as copper sulfate) 10 mg, Mn (as manganese sulfate) 20 mg, Co (as cobaltous sulfate) 0.1 mg, Zn (as zinc sulfate) 30 mg, I (as potassium iodate) 0.5 mg, Se (as sodium selenite) 0.1 mg, vitamin A 2240 IU, vitamin D_3_ 500 IU, vitamin E 40 IU, riboflavin 6.2 mg, nicotinic acid 22 mg, D-pantothenic acid 22 mg, vitamin B_12_ 0.02 mg, biotin 0.15 mg and choline 0.92 mg.

The steers were housed in individual tie stalls and fed twice daily at 06.30 and 17.30 h. Feed and orts were weighed daily to calculate feed intake. The orts were approximately 5% of the feed supplied. The steers were allowed free access to feed and water. The experiment lasted 32 days with the initial 7 days as an adaptation period. The experiment length is comparable to that of Saleem et al. [17].

### Growth performance measurements

The dry matter intake was determined for each steer daily, and the BW of each steer was measured at the beginning and end of the experiment for two consecutive days. These data were used to calculate the average daily gain (ADG), average daily feed intake (ADFI) and feed conversion ratio (ADFI/ADG).

### Sample collection

Blood samples (10 mL per steer) were collected from the jugular vein on day 20 before the morning feeding and were collected into sterile, pyrogen-free vacuum tubes containing sodium heparin (Xiamen Limulus Reagent Biotechnology Co., Ltd, Xiamen, China). During blood sampling, beef steers were kept standing and restrained from movement. Blood sampling was skillfully conducted in a gentle and quick manner to minimize the pain of the animals. Then, the blood samples were centrifuged at 4°C and 3000 × g for 15 min, and the plasma was transferred to 2-mL pyrogen-free tubes, which were immediately stored at -80°C prior to analysis.

Four pairs of beef steers were selected randomly for rumen fluid collection at the end of the experimental period. Three hours after the morning feeding, rumen fluid was collected with an oral stomach tube equipped with a 150-mL pyrogen-free syringe and a filter as described by Shen et al. [18]. When the length of the tube inserted into the rumen was approximately 200 cm, rumen fluid was obtained with a 150-mL pyrogen-free syringe. The initial 50 mL of rumen fluid was discarded to avoid saliva contamination. Subsequently, approximately 150 mL of rumen fluid was strained through four layers of sterile cheesecloth, and the filtrate was divided into two parts. One part was immediately used to determine the pH value with a portable pH meter (Rex PHS-3E, Shanghai INESA Scientific Instrument Co., Ltd., Shanghai, China), and the other part was transferred into sterile and pyrogen-free centrifuge tubes (approximately 50 mL per steer) and centrifuged at 4°C and 10,000 × g for 30 min; the supernatant was stored in 2-mL pyrogen-free tubes at -80°C prior to analysis.

### Lipopolysaccharide measurement

The LPS concentrations in the rumen fluid and plasma were determined using an ELISA kit purchased from Shanghai Preferred Biotechnology Co., Ltd. (Shanghai, China) according to the manufacturer’s instructions. All samples were tested in duplicate, and the optical density values were read at 450 nm on an automatic microplate reader (EIX808IU; BioTek, Winooski, VT, USA).

### Metabolomics analysis

The rumen fluid and plasma samples were thawed at 4°C on ice. After vortexing for 30 s, 100 μL of sample was placed in an Eppendorf tube, extracted with 300 μL of methanol and 20 μL of internal standard and vortexed for 30 s. Subsequently, the extracts were ultrasound treated for 10 min (incubated in ice water) and incubated for 1 h at -20°C to precipitate proteins. Then, the samples were centrifuged at 13,000 × g for 15 min at 4°C, and 200 μL of supernatant was transferred to LC-MS vials [19].

The LC-MS/MS analyses were performed using an UHPLC system (1290, Agilent Technologies, Santa Clara, CA, USA) with a Waters UPLC BEH Amide column (1.7 μm × 2.1 μm × 100 mm) coupled to the Triple TOF 6600 (Q-TOF, AB Sciex, Framingham, MA, USA). The mobile phase consisted of 25 mM ammonium acetate and 25 mM ammonium hydroxide in water (pH 9.75) (A) and acetonitrile (B) and was carried with the following elution gradient delivered at 0.5 mL min^-1^: 0 min, 95% B; 7 min, 65% B; 9 min, 40% B; 9.1 min, 95% B; and 12 min, 95% B. The injection volume was 2 μL. The mass spectrometer (Triple TOF 6600, AB Sciex, Framingham, MA, USA) was used to acquire MS/MS spectra according to an information-dependent acquisition (IDA). During the process, the acquisition software (Analyst TF 1.7, AB Sciex, Framingham, MA, USA) was used to evaluate the full-scan survey MS data based on preselected criteria. In each data acquisition cycle, precursor ions corresponding to an ion intensity greater than 100 were collected for fragmentation. Collision energy (CE) was 30 V (15 MS/MS events with a production accumulation time of 50 msec each). The parameters of the electron spray ionization (ESI) source were set as follows: ion source gas 1 as 60 psi, ion source gas 2 as 60 psi, curtain gas as 35 psi, source temperature 650°C, and ion spray voltage floating (ISVF) 5000 V or -4000 V in positive or negative mode, respectively [20, 21].

### Data analysis

The ADG, ADFI, ADFI/ADG, rumen pH value, and LPS concentrations of rumen fluid and plasma were analyzed by means of SAS (SAS Institute Inc., Version 9.0). Statistical significance was declared at *P* < 0.05.

MS raw data (.d) files were converted to the mzXML format using ProteoWizard (http://proteowizard.sourceforge.net/downloads.shtml) and processed with the R package XCMS (https://xcmsonline.scripps.edu/landing_page.php?pgcontent=mainPage). The preprocessing results generated a data matrix that consisted of the retention time (RT), mass to charge ratio (m/z) values, and peak intensity. The R package CAMERA was used for peak annotation after XCMS data processing. An in-house MS2 database was applied for metabolite identification [22, 23].

SIMCA software (Version 14.1, Umea, Sweden) was used in multivariate statistical analyses, including principal component analysis (PCA) and orthogonal partial least-squares discriminant analysis (OPLS-DA) [24, 25]. PCA was performed to visualize trends in the samples. To remove noise, OPLS-DA was used to explore differences between the high-corn diet (HCD) and low-corn diet (LCD) groups. Based on the OPLS-DA results, metabolites were plotted according to their importance in separating the dietary groups, and each metabolite received a value called variable importance in projection (VIP). Metabolites with VIP values exceeding 1.0 were selected as changed variables. Then, these variables were assessed using Student’s t test. If *P* < 0.05 and VIP > 1, the variable was defined as a significantly differential metabolite between the groups [26]. Using the Kyoto Encyclopedia of Genes and Genomes (KEGG, http://www.genome.jp/kegg), metabolic pathways mapped by every differential metabolite were acquired [27, 28]. Then, MetaboAnalyst (http://www.metaboanalyst.ca) was performed to analyze pathways [29], and the cutoff of impact value from the topology analysis was set to 0.1 [30].

## Results

### Growth performance

The effects of the diets on the growth performance of beef steers are presented in Table 2. The initial BW did not differ between the LCD and HCD groups, but after 25 days, a significant difference was found between the two groups in the final BW (*P* = 0.041), ADG (*P* = 0.008), ADFI (*P* = 0.008) and ADFI/ADG (*P* = 0.022).

**Table 2:** Effects of the different amounts of corn in the diet on growth performance.

Items	Treatment^a^		SEM^b^	*P*-value
	LCD	HCD		
**Initial body weight (kg)**	368.02	362.67	10.75	0.640
**Final body weight (kg)**	384.28	403.75	7.12	0.041
**Average daily gain (ADG, kg)**	0.65	1.64	0.24	0.008
**Average daily feed intake (ADFI, kg, DM)**	5.10	6.46	0.32	0.008
**Feed conversion ratio (ADFI/ADG)**	9.02	4.38	1.41	0.022

^a^LCD is the low-corn diet; HCD is the high-corn diet.

^b^Standard error of the mean.

### The ruminal pH and the rumen fluid and plasma LPS concentrations

The effects of the different diets on the ruminal pH and the rumen fluid and plasma LPS concentrations in beef cattle are presented in Table 3. For the HCD-fed beef steers, the LPS concentrations of the rumen fluid and plasma increased (*P* < 0.05), and the ruminal pH significantly decreased (*P* < 0.01) compared to those of the LCD-fed animals.

**Table 3:** Ruminal pH and the rumen fluid and plasma lipopolysaccharide (LPS) concentrations.

Items	Treatment^a^		SEM^b^	*P*-value
	LCD	HCD		
**Ruminal pH**	6.83	6.35	0.04	0.002
**LPS (rumen, EU/mL)**	21687.72	25551.89	1022.12	0.032
**LPS (plasma, EU/mL)**	0.46	0.53	0.02	0.013

^a^LCD is the low-corn diet; HCD is the high-corn diet.

^b^Standard error of the mean.

### Identification and quantification of LC-MS compounds

The ionization source of LC-QTOF/MS is electrospray ionization, including positive and negative ion modes (POS and NEG, respectively). The quality control (QC) samples were analyzed for every three samples to detect the stability and repeatability of the system. The peak retention time and peak area of total ion chromatograms (TIC) from all QC samples overlapped well, which indicated that the analytical system was stable (S2 Fig). The score scatter plot of the PCA model (S3 Fig) showed that all QC samples overlapped, which indicated the good repeatability of the analytical system.

A total of 2005 and 1770 valid peaks were identified in POS and NEG for the rumen fluid, and 1424 and 1066 valid peaks were identified in POS and NEG for the plasma, respectively. Based on an in-house MS2 database and the KEGG COMPOUND Metabolomics Library, these valid peaks were matched for 456 (POS) and 261 (NEG) rumen metabolites and for 246 (POS) and 140 (NEG) plasma metabolites.

### Multivariate analysis of rumen fluid and plasma metabolites

**Fig 1:**
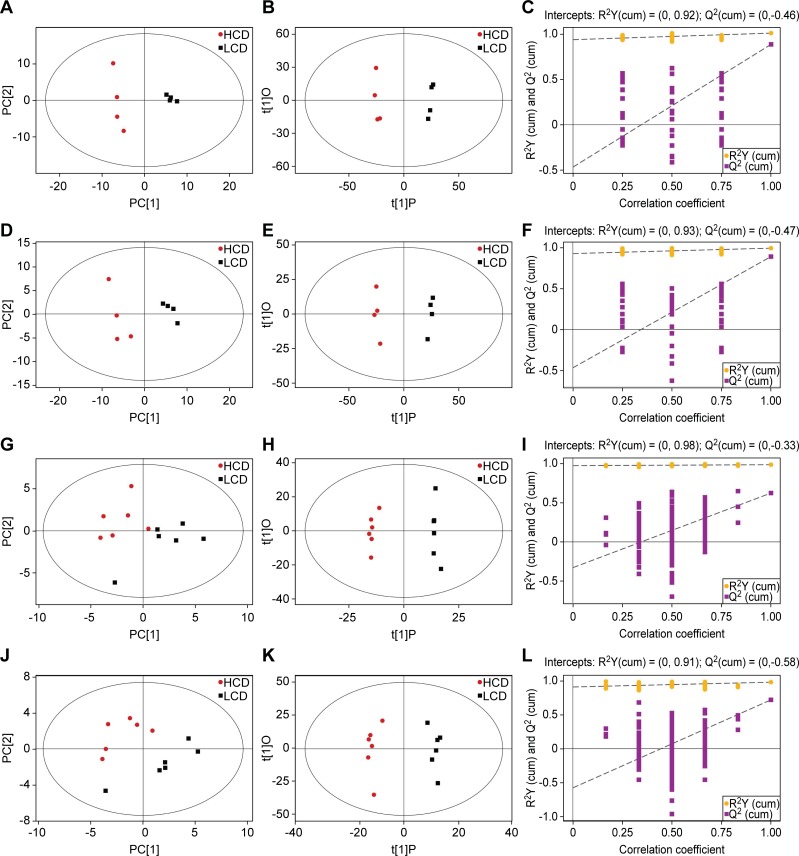
PCA model score scatter plot, OPLS-DA model and permutation test for the LCD *vs*. HCD groups. The PCA model (A, D, G and J), OPLS-DA model (B, E, H and K) and permutation test of the OPLS-DA model (C, F, I and L) were derived from the UHPLC-QTOF/MS metabolomics profiles of rumen and plasma for beef steers fed the low-corn diet (LCD) and the high-corn diet (HCD). A-C and D-F were respectively derived from POS and NEG for the rumen fluid, and G-I and J-L were respectively derived from POS and NEG for the plasma samples. Red and black respectively represent HCD and LCD administration to beef steers (A, B, D, E, G, H, J and K). Purple squares represent the *Q*^*2*^ value, and yellow dots represent the *R*^*2*^ value from the permutation test (C, F, I and L).

Because the PCA is an unsupervised pattern recognition method, it can reveal the intrinsic variation within data and reduce data dimensionality. In the PCA score scatter plot, similar datasets are clustered more closely, whereas different datasets are placed further apart. PCA of the UHPLC-QTOF/MS metabolic profiles of all samples, including the rumen fluid and plasma, is illustrated in Fig 1 (A, D, G and J). In each PCA score scatter plot, there were distinctly separated clusters between the HCD and LCD groups. All samples in each score scatter plot were within the 95% Hotelling’s T-squared ellipse. Based on these results, the metabolic datasets warranted further analysis.

The OPLS-DA results for the rumen fluid and plasma from the two dietary treatments are displayed in Fig 1B, 1E, 1H and 1K. There were very distinct separations between the LCD and HCD groups. All samples in each score scatter plot of the OPLS-DA model were inside the 95% Hotelling’s T-squared ellipse. The validity of the OPLS-DA model was evaluated using *R*^*2*^*Y* and *Q*^*2*^, in which *R*^*2*^*Y* and *Q*^*2*^ were applied to evaluate model stability and the ability to explain and predict the raw data. The closer the *R*^*2*^*Y* and *Q*^*2*^ values are to 1, the better the model should be. In this study, *R*^*2*^*Y* and *Q*^*2*^ of the rumen fluid (POS, NEG) were 0.995, 0.873 and 0.994, 0.891, respectively, and *R*^*2*^*Y* and *Q*^*2*^ of the plasma (POS, NEG) were 0.991, 0.627 and 0.980, 0.720, respectively. To avoid the transition fit of the OPLS-DA mode, the permutation test was used for verification; the results are shown in Fig 1C, 1F, 1I and 1L. The permutation test results for the *R*^*2*^*Y* and *Q*^*2*^ intercepts were 0.92 and -0.46 for the rumen fluid POS, 0.93 and -0.47 for the rumen fluid NEG, 0.98 and -0.33 for the plasma POS, and 0.91 and -0.58 for the plasma NEG. The results indicated that the OPLS-DA model had no overfitting and good stability and thus was suitable to explore the differences between the two diets in this study.

### Significantly differential metabolites between the two diets

**Fig 2:**
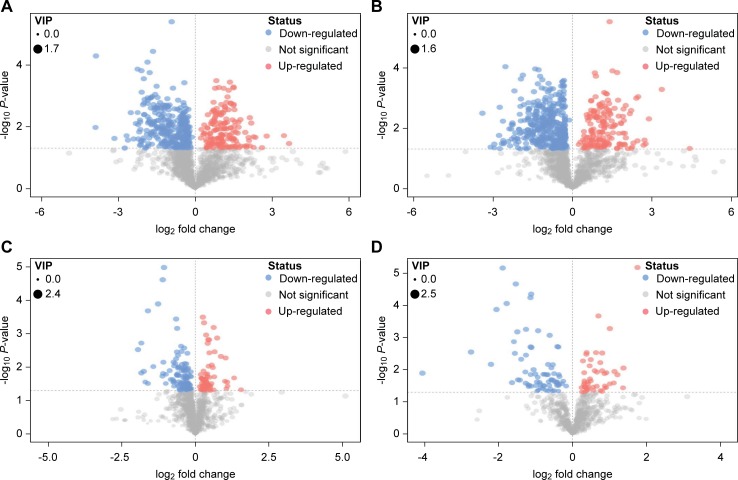
Volcano plots for the LCD *vs*. HCD groups. A and B were respectively derived from POS and NEG of the rumen fluid, and C and D were respectively derived from POS and NEG of the plasma samples. In the volcano plot, each point represents a metabolite, and the point size represents the VIP value of this metabolite in the OPLS-DA model. Compared with the LCD group, red indicates a significantly upregulated metabolite in the HCD group, whereas blue indicates the opposite, and gray shows no significant difference between the two groups.

According to the principle that the *P* value of the t-test was < 0.05 and the VIP of the OPLS-DA model was > 1, significantly differential metabolites between the two diets were screened from all identified metabolites. The significantly differential metabolites were visualized through volcano plots (Fig 2). The figure clearly shows that many metabolites in the rumen fluid and plasma samples were significantly different between the two groups.

**Fig 3:**
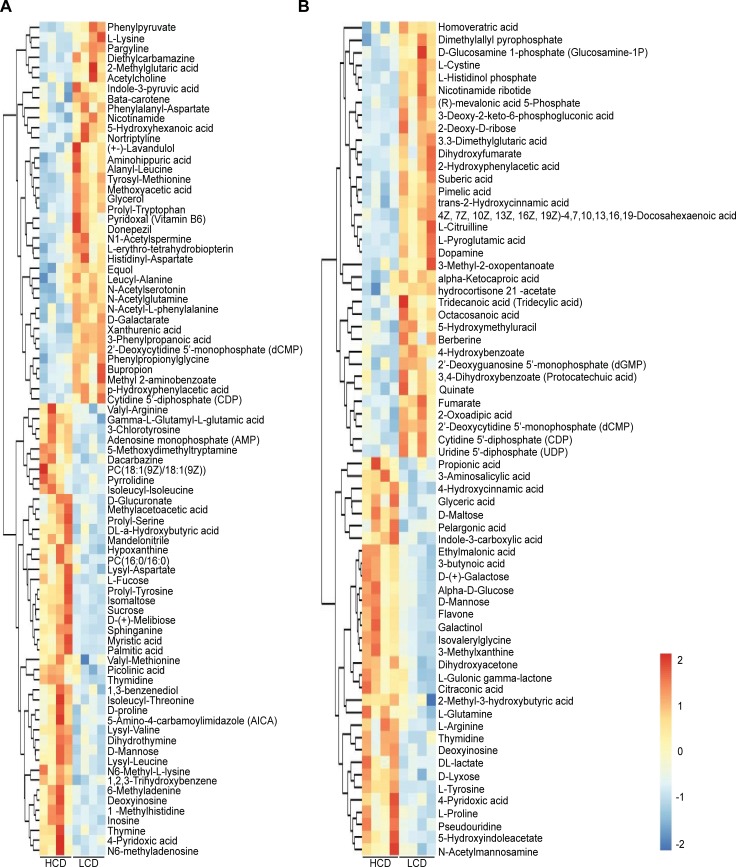
Heatmap of hierarchical clustering analysis of the differential rumen metabolites in beef cattle fed the high-corn diet (HCD) and the low-corn diet (LCD). A and B were derived from POS and NEG, respectively. Each column represents one beef steer, and each row represents one differential metabolite. The color of every cell represents the relative level of the differential metabolite, with red indicating high levels and blue low levels in rumen.

In total, 144 significantly differential metabolites were obtained in the rumen between the LCD and HCD groups (Fig 3). Compared with the LCD-fed steers, 73 significantly differential metabolites in the rumen fluid had higher concentrations in the HCD-fed steers, including carbohydrates, amino acids, organic acids, two peptides, and lipids. The concentrations of all sugars were significantly increased in the HCD group, including dihydroxyacetone (FC = 1.73), L-fucose (FC = 1.99), D-mannose (FC = 2.89), sucrose (FC = 2.35), isomaltose (FC = 2.61), D-(+)-melibiose (FC = 4.23), D-lyxose (FC = 2.02), D-(+)-galactose (FC = 2.70), alpha-D-glucose (FC = 3.74), D-maltose (FC = 2.53) and galactinol (FC = 3.34) (S1 Table). Except for L-lysine, L-citrulline and L-cystine, the concentrations of the identified amino acids were higher in the HCD group than in the LCD group. Notably, the DL-lactate (FC = 1.93), propionic acid (FC = 1.35), hypoxanthine (FC = 1.18), 1-methylhistidine (FC = 1.73), indole-3-carboxylic acid (FC = 1.60), 5-hydroxyindoleacetate (FC = 3.41), 5-methoxydimethyltryptamine (FC = 1.76), 1,2,3-trihydroxybenzene (FC = 2.24), 3-methylxanthine (FC = 2.00), N-acetylmannosamine (FC = 1.38), picolinic acid (FC = 1.99), and thymine (FC = 4.03) concentrations were also increased in the rumen fluid of the HCD-fed animals (S1 Table).

**Fig 4:**
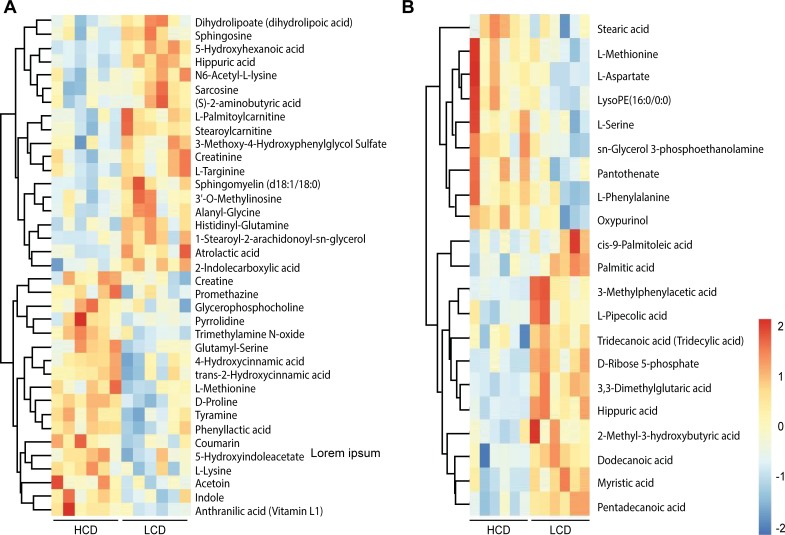
Heatmap of hierarchical clustering analysis of the differential plasma metabolites in beef cattle fed the high-corn diet (HCD) and the low-corn diet (LCD). A and B were derived from POS and NEG, respectively. Each column represents one beef steer, and each row represents one differential metabolite. The color of every cell represents the relative level of the differential metabolite, with red indicating high levels and blue low levels in plasma.

Among the 56 significantly differential metabolites in the plasma, 26 metabolites had higher concentrations in the HCD-fed steers than in the LCD-fed steers (Fig 4). The concentrations of all identified amino acids increased in the HCD-fed steers, including D-proline (FC = 1.23), L-methionine (FC = 1.17), L-lysine (FC = 1.22), L-serine (FC = 1.25), L-aspartate (FC = 1.58) and L-phenylalanine (FC = 1.24). The tyramine (FC = 1.21), indole (FC = 1.17), 5-hydroxyindoleacetate (FC = 1.17), trimethylamine N-oxide (FC = 2.04), and phenyllactic acid (FC = 1.19) concentrations were higher in the HCD-fed steers than in the LCD-fed steers (S2 Table).

### Metabolic pathway analysis of metabolites

**Fig 5:**
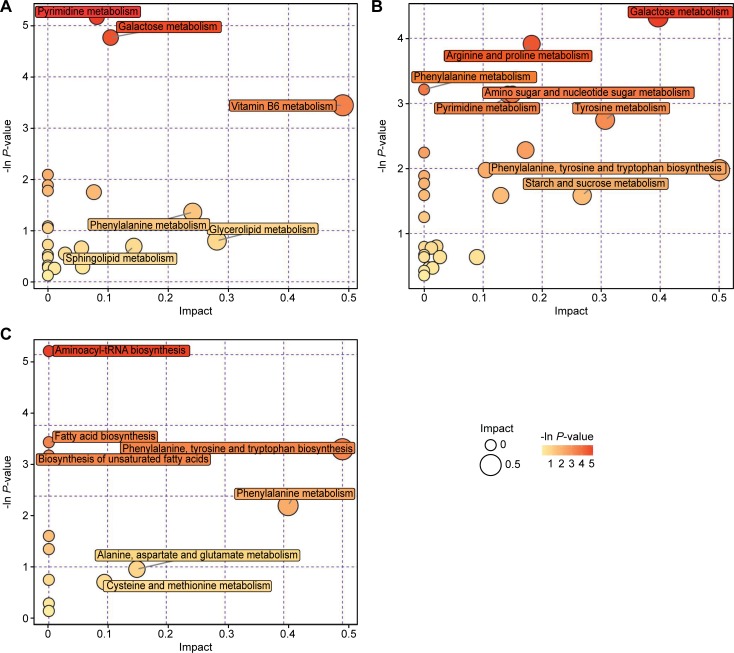
Bubble plots of the metabolic pathway analysis for the LCD *vs*. HCD groups. A and B were respectively derived from POS and NEG of the rumen fluid, and C was derived from NEG of the plasma samples. Each bubble in the bubble map represents a metabolic pathway. The x-axis represents a pathway impact value in the topology analysis, and larger bubbles represent higher pathway impact values. The y-axis represents the *P*-value (-ln*p*) of the metabolic pathway in the enrichment analysis, and the darker color of bubble represents higher pathway enrichment.

As shown in Fig 5A and 5B, the bubble plots demonstrated the main influential metabolic pathways in which the significantly differential metabolites in the rumen were involved. Fifty-three metabolic pathways were found (POS: 24; NEG: 29) (S3 Table). The impact values of five pathways in POS were greater than 0.1. The impact values of vitamin B6 metabolism; glycerolipid metabolism; phenylalanine metabolism; sphingolipid metabolism and galactose metabolism were 0.49, 0.28, 0.24, 0.14 and 0.10, respectively (S3 Table). The impact values of phenylalanine, tyrosine and tryptophan biosynthesis; galactose metabolism; tyrosine metabolism; starch and sucrose metabolism; arginine and proline metabolism; terpenoid backbone biosynthesis; pyrimidine metabolism; amino sugar and nucleotide sugar metabolism; alanine, aspartate and glutamate metabolism; and glycerolipid metabolism in NEG were 0.50, 0.40, 0.31, 0.27, 0.18, 0.17, 0.15, 0.14, 0.13 and 0.10, respectively (S3 Table).

Metabolomics pathways with impact values > 0.1 were only found in the plasma NEG (Fig 5C). The impact values of phenylalanine, tyrosine and tryptophan biosynthesis; alanine, aspartate and glutamate metabolism; and phenylalanine metabolism were 0.50, 0.15 and 0.41, respectively (S4 Table).

## Discussion

Metabolomics analyses changes in all chemical compounds or metabolites generated by cells and organisms that are stimulated or disturbed [31]. The new methodology is in its pioneering stage, and often, no perfect technology is available to identify and match all molecules. Saleem et al. [32] identified 246 rumen metabolites in dairy cows using multi-metabolomics methodologies (combined NMR, GC-MS, lipidomics, computer-aided literature and so forth). Zhang et al. [33] detected 267 endogenous plasma metabolomes in 76 dairy cows by GC/MS. However, we identified 717 and 386 metabolites in rumen fluid and plasma, respectively, using UHPLC-QTOF/MS; thus, more metabolites were identified in this experiment. UHPLC has a better separation efficiency, peak capacity and sensitivity and provides a more suitable interface for mass spectrometry, which contributes to the detection of more metabolites. For instance, we identified many secondary metabolites in this study, including dihydroxyacetone, 2-hydroxyphenylacetic acid, and 5-hydroxyhexanoic acid. The results indicate that metabolomics based on UHPLC-QTOF/MS can provide more information.

### Rumen metabolomics profiling associated with a high-corn diet

Saleem et al. [17] reported that PCA score plots revealed differences in ruminal metabolites for cows fed different amounts (0, 15, 30 and 45%) of barley grain in their diets; the clusters corresponding to the rumen metabolite profiles of the diets with 0 and 15% grain strongly overlapped with each other, whereas the clusters representing the diet with 45% grain were spaced further apart from those corresponding to the diets with 0, 15, and 35% grain. Similar to their study, this study clearly showed significant differences in rumen fluid and plasma metabolites of beef steers fed diets with different corn contents. The concentrations of sugars, two peptides and most amino acids were higher in the HCD group. Amino acids and peptides provide substrates, and sugars supply energy for the synthesis of microbial proteins (MCP) [34]. These findings suggest that beef steers may obtain more nutrients from the HCD than from the LCD. These results agree with the phenotypic measurements, including the final BW, ADG and ADFI/ADG (Table 2).

The rumen is a natural microbial fermentation chamber that digests feed particles; produces MCP, vitamins and VFAs; and thus contributes to the host’s nutrition. In beef cattle production, rumen health is important for the production of high-quality meat. However, many surveys have shown that beef cattle are often fed high-grain diets to meet the nutritional requirements of rapid growth on feedlot farms. As a result, organic acids accumulate in the rumen, and the buffering capacity of the rumen decreases and finally develops into SARA [35–37]. After grain-based SARA, many harmful or toxic substances increase in the rumen, including LPS, histamine, tyramine, tryptamine, ethanol and D-lactate [5]. In this study, the propionic acid and DL-lactate concentrations in the HCD were significantly higher than those in the LCD. The combination of these changes can lead to depression of rumen pH values. Indeed, the ruminal pH values of beef steers fed the HCD were obviously lower than those of beef steers fed the LCD in our study. These agree with the report by Zhou et al. [38], in which the ruminal pH values of cows fed an HCD were significantly lower than those of cows fed an LCD.

Gozho et al. [39] indicated that the ruminal LPS concentrations of beef cattle increased accompanying an increase in concentrate in the diet. The results of our study also showed that feeding beef steers the HCD was associated with significant increases in the rumen fluid and plasma LPS concentrations. LPS is a cell wall component of Gram-negative bacteria, and the drop in ruminal pH results in the death of Gram-negative bacteria and cell lysis [40]. According to Zhou Jun et al [38], the ruminal pH values of a low-concentrate diet and a high-concentrate diet were 6.62 and 6.21, respectively; accordingly, the LPS concentrations were 11664 EU/mL and 29065 EU/mL. Therefore, the increase in LPS concentration in this study was partly attributed to bacterial cell lysis under the lower rumen pH values. In addition to LPS, the degradation product of rumen bacteria was hypoxanthine in this study, which was higher in the HCD group. This finding was consistent with the results of Saleem et al. [17, 32], who found that the hypoxanthine concentration was elevated with increasing proportions of barley grain.

Ametaj and colleagues conducted several metabolomics experiments to reveal alterations in rumen metabolites of dairy cows fed increasing proportions of barley grain, and their studies showed that the 3-phenylpropionate (3-PP) concentration significantly decreased with the increase in barley grain [17, 32, 41]. In our study, the 3-PP concentration also significantly decreased in the HCD group (*P* < 0.001, FC = 0.07). Turlin et al. [42] indicated that the expression of some key enzymes associated with 3-PP production, such as 3-PP dioxygenase and 3-PP-2’,3’-dihydrodiol dehydrogenase, was highly inhibited in the presence of glucose. We previously reported that the glucose concentration increased significantly in HCD-fed steers.

### Plasma metabolomics profiling associated with a high-corn diet

With respect to the plasma differential metabolites, the concentrations of all amino acids quantified in this experiment increased significantly in the HCD group compared with those in the LCD group. In addition, the pantothenate content in the HCD group obviously increased. These results may partly explain why steers fed the HCD had a better growth performance.

In other studies, the nonesterified fatty acid (NEFA) concentration increased with the increase in the grain proportion in the diet [8]. The NEFA concentrations in plasma reflect the rate of adipose mobilization. Release of NEFAs in plasma is predominantly determined by their mobilization from adipose tissue triacylglycerol stores in response to variations in the energy demand of the animal. Ametaj et al. [8] noted an effect on the energy demand of cows caused by the lower dry matter intake during the night hours; as a result, the plasma NEFAs increased, especially in the low-grain and grain-free groups, early after the morning feeding. Data from this present study showed that the plasma concentrations of NEFAs, such as dodecanoic acid, tridecanoic acid, myristic acid, pentadecanoic acid and palmitic acid, decreased in the HCD-fed steers. The present study also showed that the ADFI was higher for beef steers fed the HCD, which supplied more energy, as demonstrated by their better growth performance. Therefore, we speculated that the mobilization of adipose tissue decreased in the beef cattle fed the HCD, which could lead to decreased NEFA concentrations in the plasma. Zebeli et al. [43] also demonstrated that a high feeding level did not result in a higher postpartum plasma NEFA increase in dairy cows.

### Metabolic pathways associated with a high-corn diet

The study not only identified differential metabolites between the HCD and LCD groups but also analyzed the metabolic pathways in which these metabolites were involved. Analysis of the key metabolic pathways included enrichment analysis and topology analysis by KEGG and MetaboAnalyst. Although *P*-values calculated from enrichment analysis indicate metabolic pathway enrichment, there is no standard threshold for *P*-values [44]. In this study, we explored the key metabolic pathways based on the impact values and *P*-values [30, 44, 45]. Through a comprehensive analysis of metabolic pathways in rumen fluid and plasma, we found that the common metabolic pathways of the two biofluids were phenylalanine, tyrosine and tryptophan biosynthesis and phenylalanine metabolism. Phenylalanine, tyrosine and tryptophan are aromatic essential amino acids. The two metabolic pathways were associated with phenylalanine, demonstrating that phenylalanine played a vital role in relation to metabolic pathways of the high-starch diet. Not only was L-phenylalanine significantly different between the two diets, but also many metabolites involved in phenylalanine metabolism were significantly different, including phenyllactic acid and tyramine in the plasma. The main metabolic pathway of phenylalanine is tyrosine production by catalysis of phenylalanine hydroxylase. Tyramine is produced during the metabolic process of tyrosine. In addition, phenylalanine is converted to phenyllactic acid through phenylpyruvate. In this study, phenyllactic acid and tyramine were higher in the plasma of the HCD group. The effect of phenyllactic acid on ruminant health has not been reported at present. However, tyramine is a biological amine, and large amounts of tyramine prevent epithelium regeneration and induce epithelial injury. The study by Mao et al. also showed that some toxic, inflammatory and abnormal metabolites increased with increasing dietary corn grain, such as LPS, tryptamine, tyramine, histamine and phenylacetate [46]. In addition, our another unpublished study on the effect of the treatment of corn in a high-corn diet with 1% lactic acid on plasma metabolomics in beef steers revealed that the treatment reduced the plasma relative abundance of phenylalanine (VIP = 2.36, *P* < 0.001, FC = 0.76), tyramine (VIP = 2.39, *P* < 0.001, FC = 0.75) and phenyllactic acid (VIP = 2.30, *P* = 0.003, FC = 0.79). Collectively, these results indicated that phenylalanine metabolism was the common key metabolic pathway involved in a high-corn diet.

## Conclusions

When steers were fed the HCD, rumen and plasma carbohydrate metabolites and amino acids increased, which contributed to the growth of the beef steers. However, with increasing amounts of corn in the diet, the rumen acidity and rumen and plasma LPS concentrations also significantly increased. In addition, some potentially harmful metabolites, such as lactic acid in the rumen and tyramine in the plasma, increased when the beef steers were fed high-corn diet. This study also demonstrated that phenylalanine, tyrosine and tryptophan biosynthesis and phenylalanine metabolism were the common key metabolic pathways involved in a high-corn diet in both biofluids.

## Supporting information

S1 Table Identification of significantly differential metabolites in rumen fluid between the LCD and HCD groups. (DOCX)

S2 Table Identification of significantly differential metabolites in plasma between the LCD and HCD groups. (DOCX)

S3 Table Metabolic pathway analysis in rumen. (DOCX)

S4 Table Metabolic pathway analysis in plasma. (DOCX)

S1 Fig Score scatter plot of PCA model for baseline metabolomics analyses of 24 beef steers for selecting the experimental animals. (PDF)

S2 Fig TIC diagram of QC samples. (PDF)

S3 Fig Score scatter plot of the PCA model. (PDF)
