# Supplementary material for: Rumen and plasma metabolomics profiling by UHPLC-QTOF/MS revealed metabolic alterations associated with a high-corn diet in beef steers
Source: PLoS One. 2018 Nov 28;13(11):e0208031. doi: 10.1371/journal.pone.0208031 (PMC6261619; doi:10.1371/journal.pone.0208031)
Supplement: S1 Table — (DOCX) [file pone.0208031.s001.docx]

**S1 Table. Identification of significantly differential metabolites in rumen fluid between the LCD and HCD groups^a^**

| Metabolites | VIP^b^ | *P* value^c^ | FC^d^ | Metabolites | VIP | *P*  value | FC |
| --- | --- | --- | --- | --- | --- | --- | --- |
| (+-)-Lavandulol | 1.41 | 0.025 | 0.58 | Hydrocortisone 21-acetate | 1.13 | 0.033 | 0.69 |
| 4,7,10,13,1,6,19-Docosahexaenoic acid | 1.48 | 0.003 | 0.68 | Hypoxanthine | 1.43 | 0.012 | 1.18 |
| (R)-mevalonic acid 5-phosphate | 1.44 | 0.007 | 0.46 | Indole-3-carboxylic acid | 1.43 | 0.007 | 1.60 |
| 1,2,3-Trihydroxybenzene | 1.46 | 0.002 | 2.24 | Indole-3-pyruvic acid | 1.32 | 0.025 | 0.73 |
| 1,3-Benzenediol | 1.40 | 0.024 | 1.16 | Inosine | 1.57 | 0.004 | 1.94 |
| 1-Methylhistidine | 1.52 | 0.032 | 1.73 | Isoleucyl-isoleucine | 1.32 | 0.025 | 1.58 |
| 2'-Deoxycytidine 5'-monophosphate | 1.67 | <0.001 | 0.53 | Isoleucyl-threonine | 1.38 | 0.037 | 1.90 |
| 2'-Deoxy-D-ribose | 1.48 | 0.039 | 0.20 | Isomaltose | 1.64 | 0.002 | 2.61 |
| 2'-Deoxyguanosine 5'-monophosphate | 1.16 | 0.047 | 0.69 | Isovalerylglycine | 1.45 | 0.004 | 1.93 |
| 2-Hydroxyphenylacetic acid | 1.38 | 0.011 | 0.76 | L-arginine | 1.19 | 0.032 | 1.60 |
| 2-Methyl-3-hydroxybutyric acid | 1.14 | 0.036 | 1.17 | L-citrulline | 1.48 | 0.006 | 0.59 |
| 2-Methylglutaric acid | 1.53 | 0.038 | 0.47 | L-cystine | 1.53 | <0.001 | 0.40 |
| 2-Oxoadipic acid | 1.41 | 0.008 | 0.82 | L-erythro-tetrahydrobiopterin | 1.30 | 0.023 | 0.75 |
| 3,3-Dimethylglutaric acid | 1.41 | 0.009 | 0.47 | Leucyl-alanine | 1.10 | 0.025 | 0.54 |
| 3,4-Dihydroxybenzoate | 1.30 | 0.026 | 0.47 | L-fucose | 1.50 | 0.009 | 1.99 |
| 3-Aminosalicylic acid | 1.38 | 0.005 | 1.82 | L-glutamine | 1.03 | 0.038 | 2.58 |
| 3-Butynoic acid | 1.43 | 0.003 | 2.61 | L-gulonic gamma-lactone | 1.21 | 0.037 | 1.71 |
| 3-Chlorotyrosine | 1.64 | <0.001 | 1.89 | L-histidinol phosphate | 1.59 | <0.001 | 0.45 |
| 3-Deoxy-2-keto-6-phosphogluconic acid | 1.52 | 0.021 | 0.38 | L-lysine | 1.43 | 0.048 | 0.58 |
| 3-Methyl-2-oxopentanoate | 1.33 | 0.033 | 0.46 | L-proline | 1.25 | 0.044 | 1.92 |
| 3-Methylxanthine | 1.47 | 0.004 | 2.00 | L-pyroglutamic acid | 1.55 | 0.013 | 0.28 |
| 3-Phenylpropanoic acid (3-PP) | 1.59 | <0.001 | 0.07 | L-tyrosine | 1.60 | 0.001 | 5.32 |
| 4-Hydroxybenzoate | 1.42 | 0.003 | 0.49 | Lysyl-aspartate | 1.24 | 0.049 | 1.36 |
| 4-Hydroxycinnamic acid | 1.39 | 0.005 | 1.57 | Lysyl-leucine | 1.55 | 0.007 | 1.70 |
| 4-Pyridoxic acid | 1.40 | 0.029 | 3.01 | Lysyl-valine | 1.54 | 0.003 | 1.78 |
| 5-Amino-4-carbamoylimidazole | 1.41 | 0.026 | 1.34 | Mandelonitrile | 1.57 | 0.004 | 1.89 |
| 5-Hydroxyhexanoic acid | 1.54 | 0.004 | 0.42 | Methoxyacetic acid | 1.59 | 0.001 | 0.34 |
| 5-Hydroxyindoleacetate | 1.47 | 0.035 | 3.41 | Methyl 2-aminobenzoate | 1.31 | 0.034 | 0.49 |
| 5-Hydroxymethyluracil | 1.26 | 0.019 | 0.81 | Methylacetoacetic acid | 1.33 | 0.036 | 1.92 |
| 5-Methoxydimethyltryptamine | 1.51 | 0.004 | 1.76 | Myristic acid | 1.57 | 0.042 | 4.37 |
| 6-Methyladenine | 1.52 | 0.012 | 2.19 | N1-acetylspermine | 1.30 | 0.031 | 0.53 |
| Acetylcholine | 1.24 | 0.046 | 0.46 | N6-methyladenosine | 1.53 | 0.043 | 2.92 |
| Adenosine monophosphate | 1.60 | <0.001 | 2.46 | N6-methyl-L-lysine | 1.55 | 0.004 | 2.13 |
| Alanyl-leucine | 1.39 | 0.018 | 0.51 | N-acetylglutamine | 1.48 | 0.002 | 0.67 |
| Alpha-D-glucose | 1.33 | 0.015 | 3.74 | N-acetyl-L-phenylalanine | 1.35 | 0.002 | 0.25 |
| Alpha-ketocaproic acid | 1.27 | 0.016 | 0.67 | N-acetylmannosamine | 1.26 | 0.042 | 1.38 |
| Aminohippuric acid | 1.28 | 0.021 | 0.58 | N-acetylserotonin | 1.14 | 0.036 | 0.46 |
| Bata-carotene | 1.20 | 0.010 | 0.45 | Nicotinamide | 1.33 | 0.015 | 0.49 |
| Berberine | 1.14 | 0.046 | 0.72 | Nicotinamide ribotide | 1.57 | 0.008 | 0.37 |
| Bupropion | 1.46 | 0.014 | 0.43 | Nortriptyline | 1.46 | 0.008 | 0.69 |
| Citraconic acid | 1.24 | 0.033 | 2.31 | Octacosnoic acid | 1.45 | 0.004 | 0.48 |
| Cytidine 5'-diphosphate | 1.44 | 0.008 | 0.20 | Palmitic acid | 1.61 | 0.020 | 6.90 |
| D-(+)-galactose | 1.45 | 0.004 | 2.70 | Pargyline | 1.46 | 0.008 | 0.31 |
| D-(+)-melibiose | 1.61 | 0.023 | 4.23 | PC(16:0/16:0) | 1.48 | 0.009 | 1.67 |
| Dacarbazine | 1.33 | 0.017 | 1.26 | PC(18:1(9Z)/18:1(9Z)) | 1.49 | 0.046 | 2.59 |
| Deoxyinosine | 1.56 | 0.008 | 2.25 | Pelargonic acid | 1.23 | 0.035 | 1.58 |
| D-galactarate | 1.47 | 0.002 | 0.43 | Phenylalanyl-aspartate | 1.36 | 0.028 | 0.80 |
| D-glucosamine 1-phosphate | 1.40 | 0.020 | 0.44 | Phenylpropionylglycine | 1.31 | 0.017 | 0.67 |
| D-glucuronate | 1.40 | 0.049 | 1.36 | Phenylpyruvate | 1.42 | 0.012 | 0.43 |
| Diethylcarbamazine | 1.55 | 0.003 | 0.49 | p-hydroxyphenylacetic acid | 1.59 | 0.002 | 0.48 |
| Dihydrothymine | 1.42 | 0.025 | 2.43 | Picolinic acid | 1.23 | 0.025 | 1.99 |
| Dihydroxyacetone | 1.35 | 0.011 | 1.73 | Pimelic acid | 1.45 | 0.002 | 0.69 |
| Dihydroxyfumarate | 1.10 | 0.046 | 0.13 | Prolyl-serine | 1.42 | 0.025 | 1.52 |
| Dimethylallyl pyrophosphate | 1.25 | 0.009 | 0.49 | Prolyl-tryptophan | 1.55 | 0.001 | 0.47 |
| DL-a-hydroxybutyric acid | 1.48 | 0.007 | 1.67 | Prolyl-tyrosine | 1.60 | 0.018 | 1.99 |
| DL-lactate | 1.39 | 0.041 | 1.93 | Propionic acid | 1.21 | 0.038 | 1.35 |
| D-lyxose | 1.42 | 0.002 | 2.02 | Pseudouridine | 1.29 | 0.038 | 2.34 |
| D-maltose | 1.52 | 0.001 | 2.53 | Pyridoxal (vitamin B6) | 1.33 | 0.035 | 0.50 |
| D-mannose | 1.38 | 0.009 | 2.89 | Pyrrolidine | 1.25 | 0.048 | 2.97 |
| Donepezil | 1.41 | 0.020 | 0.16 | Quinate | 1.29 | 0.020 | 0.54 |
| Dopamine | 1.58 | 0.018 | 0.15 | Sphinganine | 1.64 | 0.008 | 4.54 |
| D-proline | 1.41 | 0.023 | 1.52 | Suberic acid | 1.47 | 0.003 | 0.57 |
| Equol | 1.20 | 0.035 | 0.74 | Sucrose | 1.65 | 0.007 | 2.35 |
| Ethylmalonic acid | 1.44 | 0.003 | 1.58 | Thymidine | 1.34 | 0.018 | 2.29 |
| Flavone | 1.50 | 0.002 | 2.04 | Thymine | 1.32 | 0.041 | 4.03 |
| Fumarate | 1.53 | 0.011 | 0.49 | Trans-2-hydroxycinnamic acid | 1.40 | 0.002 | 0.55 |
| Galactinol | 1.51 | 0.004 | 3.34 | Tridecanoic acid | 1.25 | 0.041 | 0.56 |
| Gamma-L-glutamyl-L-glutamic acid | 1.45 | 0.005 | 1.70 | Tyrosyl-methionine | 1.50 | 0.003 | 0.43 |
| Glyceric acid | 1.32 | 0.021 | 2.23 | Uridine 5'-diphosphate | 1.37 | 0.025 | 0.47 |
| Glycerol | 1.62 | 0.001 | 0.55 | Valyl-arginine | 1.33 | 0.019 | 1.39 |
| Histidinyl-aspartate | 1.44 | 0.013 | 0.48 | Valyl-methionine | 1.06 | 0.035 | 1.81 |
| Homoveratric acid | 1.15 | 0.016 | 0.50 | Xanthurenic acid | 1.47 | <0.001 | 0.21 |

^a^LCD is the low-corn diet; HCD is the high-corn diet.

^b^Variable importance in the projection (VIP) was obtained from OPLS-DA model with value higher than 1.0.

^c^The *P* value calculated from two-tailed student’s test.

^d^Fold change (FC): Mean value of peak area obtained from the HCD group/Mean value of peak area obtained from the LCD group. If the FC value was larger than 1, it meant that a metabolite was more in HCD than in LCD.
