# Supplementary material for: Rumen and plasma metabolomics profiling by UHPLC-QTOF/MS revealed metabolic alterations associated with a high-corn diet in beef steers
Source: PLoS One. 2018 Nov 28;13(11):e0208031. doi: 10.1371/journal.pone.0208031 (PMC6261619; doi:10.1371/journal.pone.0208031)
Supplement: S2 Table — (DOCX) [file pone.0208031.s002.docx]

**S2 Table.** **Identification of significantly differential metabolites in plasma between the LCD and HCD groups^a^**

| Metabolites | VIP^b^ | *P* value^c^ | FC^d^ | Metabolites | VIP | *P* value | FC |
| --- | --- | --- | --- | --- | --- | --- | --- |
| (S)-2-aminobutyric acid | 1.64 | 0.031 | 0.73 | L-aspartate | 1.99 | 0.012 | 1.58 |
| 1-Stearoyl-2-arachidonoyl-sn-glycerol | 1.71 | 0.009 | 0.61 | L-lysine | 1.59 | 0.035 | 1.22 |
| 2-Indolecarboxylic acid | 1.87 | 0.002 | 0.72 | L-methionine | 1.52 | 0.036 | 1.17 |
| 2-Methyl-3-hydroxybutyric acid | 1.71 | 0.040 | 0.66 | L-palmitoylcarnitine | 1.97 | 0.003 | 0.72 |
| 3,3-Dimethylglutaric acid | 2.39 | <0.001 | 0.35 | L-phenylalanine | 1.56 | 0.029 | 1.24 |
| 3-Methoxy-4-hydroxyphenylglycol Sulfate | 1.46 | 0.046 | 0.76 | L-pipecolic acid | 1.73 | 0.029 | 0.78 |
| 3-Methylphenylacetic acid | 2.21 | 0.014 | 0.50 | L-serine | 1.65 | 0.022 | 1.25 |
| 3'-O-methylinosine | 1.64 | 0.028 | 0.61 | L-targinine | 1.78 | 0.018 | 0.76 |
| 4-Hydroxycinnamic acid | 1.68 | 0.024 | 1.22 | LysoPE(16:0/0:0) | 1.82 | 0.019 | 1.39 |
| 5-Hydroxyhexanoic acid | 2.42 | <0.001 | 0.42 | Myristic acid | 2.15 | 0.002 | 0.75 |
| 5-Hydroxyindoleacetate | 1.56 | 0.048 | 1.17 | N6-acetyl-L-lysine | 1.75 | 0.018 | 0.68 |
| Acetoin | 1.48 | 0.037 | 2.13 | Oxypurinol | 1.91 | 0.005 | 1.22 |
| Alanyl-glycine | 1.65 | 0.023 | 0.56 | Palmitic acid | 1.85 | 0.009 | 0.67 |
| Anthranilic acid (vitamin L1) | 2.09 | 0.005 | 1.83 | Pantothenate | 1.71 | 0.034 | 1.26 |
| Atrolactic acid | 1.93 | 0.015 | 0.47 | Pentadecanoic acid | 2.26 | <0.001 | 0.46 |
| Cis-9-palmitoleic acid | 1.59 | 0.039 | 0.57 | Phenyllactic acid | 1.69 | 0.023 | 1.19 |
| Coumarin | 1.66 | 0.022 | 1.23 | Promethazine | 1.53 | 0.039 | 1.34 |
| Creatine | 1.57 | 0.033 | 1.36 | Pyrrolidine | 1.72 | 0.048 | 1.54 |
| Creatinine | 1.66 | 0.033 | 0.83 | Sarcosine | 1.78 | 0.013 | 0.77 |
| Dihydrolipoate | 1.74 | 0.012 | 0.84 | Sn-glycerol 3-phosphoethanolamine | 1.72 | 0.026 | 1.25 |
| Dodecanoic acid | 2.02 | 0.002 | 0.77 | Sphingomyelin (d18:1/18:0) | 1.94 | 0.008 | 0.62 |
| D-proline | 2.16 | <0.001 | 1.23 | Sphingosine | 1.70 | 0.018 | 0.78 |
| D-ribose 5-phosphate | 2.18 | <0.001 | 0.42 | Stearic acid | 1.09 | 0.037 | 1.83 |
| Glutamyl-serine | 1.76 | 0.016 | 1.28 | Stearoylcarnitine | 2.11 | <0.001 | 0.65 |
| Glycerophosphocholine | 1.45 | 0.047 | 1.13 | Trans-2-hydroxycinnamic acid | 1.67 | 0.026 | 1.24 |
| Hippuric acid | 2.42 | <0.001 | 0.48 | Tridecanoic acid | 1.61 | 0.014 | 0.59 |
| Histidinyl-glutamine | 1.90 | 0.004 | 0.83 | Trimethylamine N-oxide | 2.01 | 0.005 | 2.04 |
| Indole | 1.63 | 0.03 | 1.17 | Tyramine | 1.78 | 0.013 | 1.21 |

^a^LCD is the low-corn diet; HCD is the high-corn diet.

^b^Variable importance in the projection (VIP) was obtained from OPLS-DA model with value higher than 1.0.

^c^The *P* value calculated from two-tailed student’s test.

^d^Fold change (FC): Mean value of peak area obtained from the HCD group/Mean value of peak area obtained from the LCD group. If the FC value was larger than 1, it meant that a metabolite was more in HCD than in LCD.
