# Supplementary material for: Rumen and plasma metabolomics profiling by UHPLC-QTOF/MS revealed metabolic alterations associated with a high-corn diet in beef steers
Source: PLoS One. 2018 Nov 28;13(11):e0208031. doi: 10.1371/journal.pone.0208031 (PMC6261619; doi:10.1371/journal.pone.0208031)
Supplement: S3 Table — (DOCX) [file pone.0208031.s003.docx]

**S3 Table. Metabolic pathway analysis in rumen**

| Pathway | Hits^a^ | Raw *P*^b^ | -ln(*p*) | Impact^c^ |
| --- | --- | --- | --- | --- |
| Positive-ion mode |  |  |  |  |
| Vitamin B6 metabolism | 2 | 0.03 | 3.36 | 0.49 |
| Glycerolipid metabolism | 1 | 0.46 | 0.77 | 0.28 |
| Phenylalanine metabolism | 1 | 0.27 | 1.32 | 0.24 |
| Sphingolipid metabolism | 1 | 0.52 | 0.66 | 0.14 |
| Galactose metabolism | 4 | 0.01 | 4.61 | 0.10 |
| Pyrimidine metabolism | 5 | 0.01 | 4.98 | 0.08 |
| Purine metabolism | 5 | 0.08 | 2.59 | 0.08 |
| Tryptophan metabolism | 1 | 0.76 | 0.27 | 0.06 |
| Pyruvate metabolism | 1 | 0.53 | 0.63 | 0.06 |
| Glycolysis or Gluconeogenesis | 1 | 0.59 | 0.52 | 0.03 |
| Arginine and proline metabolism | 1 | 0.78 | 0.24 | 0.01 |
| Phenylalanine, tyrosine and tryptophan biosynthesis | 1 | 0.13 | 2.05 | 0.00 |
| Fructose and mannose metabolism | 2 | 0.13 | 2.02 | 0.00 |
| Biotin metabolism | 1 | 0.16 | 1.84 | 0.00 |
| Starch and sucrose metabolism | 2 | 0.18 | 1.71 | 0.00 |
| Amino sugar and nucleotide sugar metabolism | 2 | 0.36 | 1.03 | 0.00 |
| Fatty acid biosynthesis | 2 | 0.37 | 0.99 | 0.00 |
| Propanoate metabolism | 1 | 0.50 | 0.69 | 0.00 |
| Fatty acid elongation in mitochondria | 1 | 0.61 | 0.50 | 0.00 |
| Glycerophospholipid metabolism | 1 | 0.63 | 0.45 | 0.00 |
| Fatty acid metabolism | 1 | 0.74 | 0.30 | 0.00 |
| Tyrosine metabolism | 1 | 0.77 | 0.26 | 0.00 |
| Biosynthesis of unsaturated fatty acids | 1 | 0.77 | 0.26 | 0.00 |
| Aminoacyl-tRNA biosynthesis | 1 | 0.89 | 0.11 | 0.00 |
| Negative-ion mode |  |  |  |  |
| Phenylalanine, tyrosine and tryptophan biosynthesis | 1 | 0.14 | 1.96 | 0.50 |
| Galactose metabolism | 4 | 0.01 | 4.26 | 0.40 |
| Tyrosine metabolism | 4 | 0.07 | 2.69 | 0.31 |
| Starch and sucrose metabolism | 2 | 0.21 | 1.55 | 0.27 |
| Arginine and proline metabolism | 5 | 0.02 | 3.84 | 0.18 |
| Terpenoid backbone biosynthesis | 2 | 0.11 | 2.25 | 0.17 |
| Pyrimidine metabolism | 4 | 0.05 | 3.08 | 0.15 |
| Amino sugar and nucleotide sugar metabolism | 4 | 0.05 | 3.08 | 0.14 |
| Alanine, aspartate and glutamate metabolism | 2 | 0.21 | 1.55 | 0.13 |
| Glycerolipid metabolism | 2 | 0.14 | 1.94 | 0.10 |
| Lysine degradation | 1 | 0.54 | 0.62 | 0.09 |
| Citrate cycle (TCA cycle) | 1 | 0.54 | 0.62 | 0.03 |
| Tryptophan metabolism | 2 | 0.46 | 0.78 | 0.02 |
| Glutathione metabolism | 1 | 0.63 | 0.46 | 0.01 |
| Purine metabolism | 4 | 0.25 | 1.40 | 0.01 |
| Glycolysis or Gluconeogenesis | 1 | 0.63 | 0.46 | 0.01 |
| Phenylalanine metabolism | 2 | 0.04 | 3.18 | 0.00 |
| Ubiquinone and other terpenoid-quinone biosynthesis | 1 | 0.11 | 2.22 | 0.00 |
| Fructose and mannose metabolism | 2 | 0.16 | 1.85 | 0.00 |
| D-Glutamine and D-glutamate metabolism | 1 | 0.17 | 1.75 | 0.00 |
| Aminoacyl-tRNA biosynthesis | 4 | 0.21 | 1.54 | 0.00 |
| Ascorbate and aldarate metabolism | 1 | 0.29 | 1.23 | 0.00 |
| Vitamin B6 metabolism | 1 | 0.29 | 1.23 | 0.00 |
| Nitrogen metabolism | 1 | 0.29 | 1.23 | 0.00 |
| Glyoxylate and dicarboxylate metabolism | 1 | 0.46 | 0.78 | 0.00 |
| Pentose phosphate pathway | 1 | 0.52 | 0.66 | 0.00 |
| Propanoate metabolism | 1 | 0.54 | 0.62 | 0.00 |
| Cysteine and methionine metabolism | 1 | 0.66 | 0.42 | 0.00 |
| Glycine, serine and threonine metabolism | 1 | 0.71 | 0.34 | 0.00 |

^a^Hits is the number of significantly differential metabolites in one pathway.

^b^Raw *P* is *P* value calculated from the pathway enrichment analysis.

^c^Impact represents impact value in the pathway topology analysis.
