# Supplementary material for: Rumen and plasma metabolomics profiling by UHPLC-QTOF/MS revealed metabolic alterations associated with a high-corn diet in beef steers
Source: PLoS One. 2018 Nov 28;13(11):e0208031. doi: 10.1371/journal.pone.0208031 (PMC6261619; doi:10.1371/journal.pone.0208031)
Supplement: S4 Table — (DOCX) [file pone.0208031.s004.docx]

**S4 Table. Metabolic pathway analysis in plasma**

| Pathway | Hits^a^ | Raw *p*^b^ | -ln(*p*) | Impact^c^ |
| --- | --- | --- | --- | --- |
| Positive-ion mode |  |  |  |  |
| Cysteine and methionine metabolism | 1 | 0.34 | 1.09 | 0.09 |
| Glycine, serine and threonine metabolism | 2 | 0.08 | 2.59 | 0.06 |
| Sphingolipid metabolism | 1 | 0.26 | 1.33 | 0.05 |
| Tyrosine metabolism | 1 | 0.46 | 0.78 | 0.03 |
| Glycerophospholipid metabolism | 1 | 0.35 | 1.06 | 0.02 |
| Arginine and proline metabolism | 2 | 0.13 | 2.04 | 0.02 |
| Tryptophan metabolism | 1 | 0.45 | 0.79 | 0.02 |
| Biotin metabolism | 1 | 0.07 | 2.66 | 0.00 |
| Aminoacyl-tRNA biosynthesis | 2 | 0.23 | 1.45 | 0.00 |
| Fatty acid metabolism | 1 | 0.44 | 0.83 | 0.00 |
| Negative-ion mode |  |  |  |  |
| Phenylalanine, tyrosine and tryptophan biosynthesis | 1 | 0.03 | 3.66 | 0.50 |
| Phenylalanine metabolism | 1 | 0.06 | 2.87 | 0.41 |
| Alanine, aspartate and glutamate metabolism | 1 | 0.14 | 1.97 | 0.15 |
| Cysteine and methionine metabolism | 1 | 0.17 | 1.79 | 0.09 |
| Aminoacyl-tRNA biosynthesis | 3 | 0.01 | 5.05 | 0.00 |
| Fatty acid biosynthesis | 2 | 0.02 | 3.76 | 0.00 |
| Biosynthesis of unsaturated fatty acids | 2 | 0.03 | 3.58 | 0.00 |
| Histidine metabolism | 1 | 0.09 | 2.44 | 0.00 |
| beta-Alanine metabolism | 1 | 0.11 | 2.25 | 0.00 |
| Fatty acid elongation in mitochondria | 1 | 0.16 | 1.82 | 0.00 |
| Fatty acid metabolism | 1 | 0.23 | 1.49 | 0.00 |
| Arginine and proline metabolism | 1 | 0.25 | 1.38 | 0.00 |

^a^Hits is the number of significantly differential metabolites in one pathway.

^b^Raw *P* is *P* value calculated from the pathway enrichment analysis.

^c^Impact represents impact value in the pathway topology analysis.
