# Supplementary material for: Rumen and plasma metabolomics profiling by UHPLC-QTOF/MS revealed metabolic alterations associated with a high-corn diet in beef steers
Source: PLoS One. 2018 Nov 28;13(11):e0208031. doi: 10.1371/journal.pone.0208031 (PMC6261619; doi:10.1371/journal.pone.0208031)
Supplement: S1 Fig — (PDF) [file pone.0208031.s005.pdf]

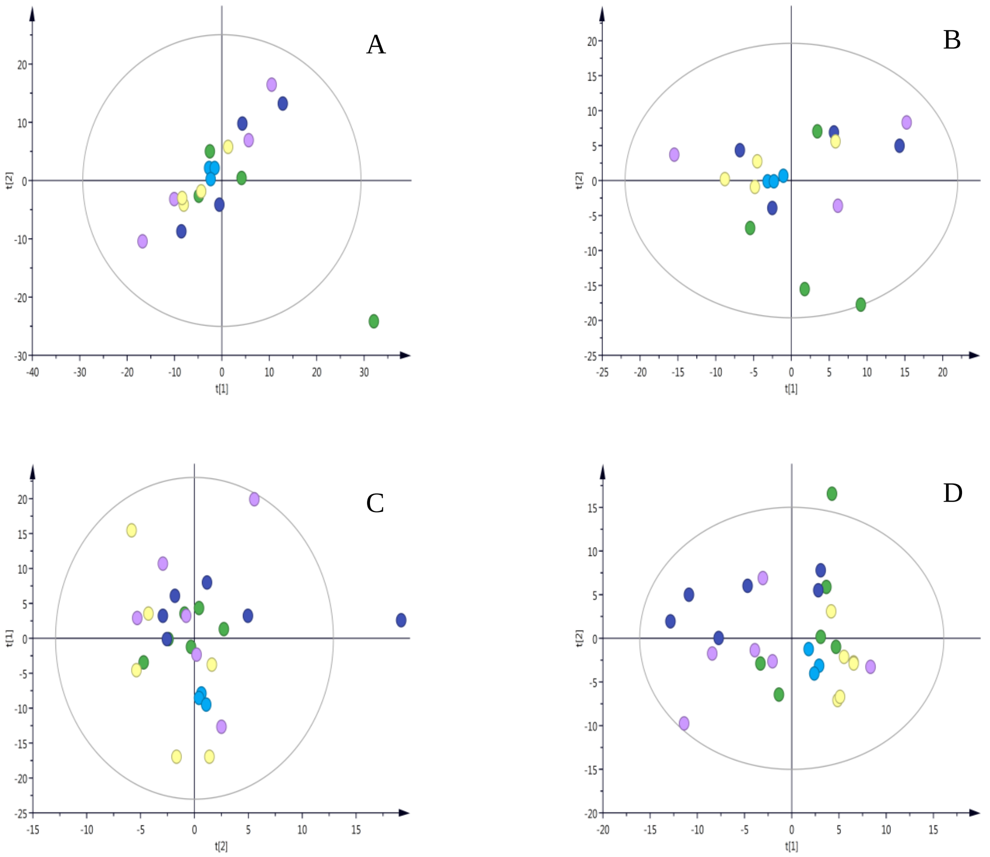

S1 Fig. Score scatter plot of PCA model for baseline metabolomics analyses of 24 beef steers for selecting the experimental animals. A and B were respectively derived from POS and NEG of the rumen fluid, and C and D were respectively derived from POS and NEG of the plasma samples. Yellow dots represent beef cattle Nos. 1-6, green dots beef cattle Nos. 7-12, purple dots beef cattle Nos. 13-18, dark blue dots beef cattle Nos. 19-24, and light blue dots the quality control (QC) samples. R^2X values of A and B were 0.492 and 0.409, respectively; and R^2X values of C and D were 0.357 and 0.355, respectively.
