# Supplementary figures and images for: Rumen and plasma metabolomics profiling by UHPLC-QTOF/MS revealed metabolic alterations associated with a high-corn diet in beef steers
Source: PLoS One. 2018 Nov 28;13(11):e0208031. doi: 10.1371/journal.pone.0208031 (PMC6261619; doi:10.1371/journal.pone.0208031)

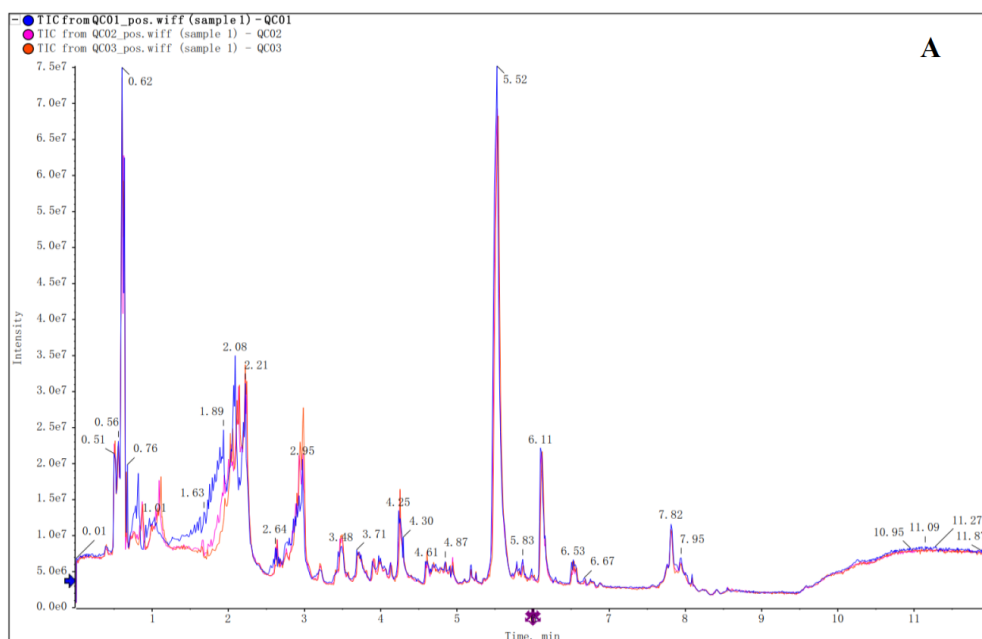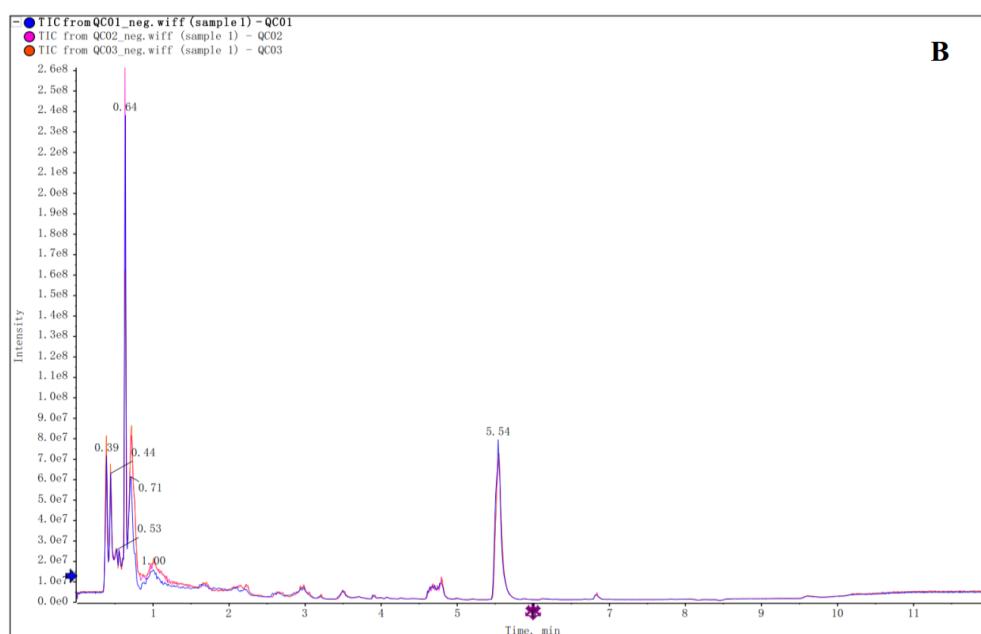

**S2 Fig. Tic diagram of QC samples.** A was derived from POS and B was derived from NEG.

Supplement: S2 Fig — (PDF) [file pone.0208031.s006.pdf]
