# Supplementary material for: Rumen and plasma metabolomics profiling by UHPLC-QTOF/MS revealed metabolic alterations associated with a high-corn diet in beef steers
Source: PLoS One. 2018 Nov 28;13(11):e0208031. doi: 10.1371/journal.pone.0208031 (PMC6261619; doi:10.1371/journal.pone.0208031)
Supplement: S3 Fig — (PDF) [file pone.0208031.s007.pdf]

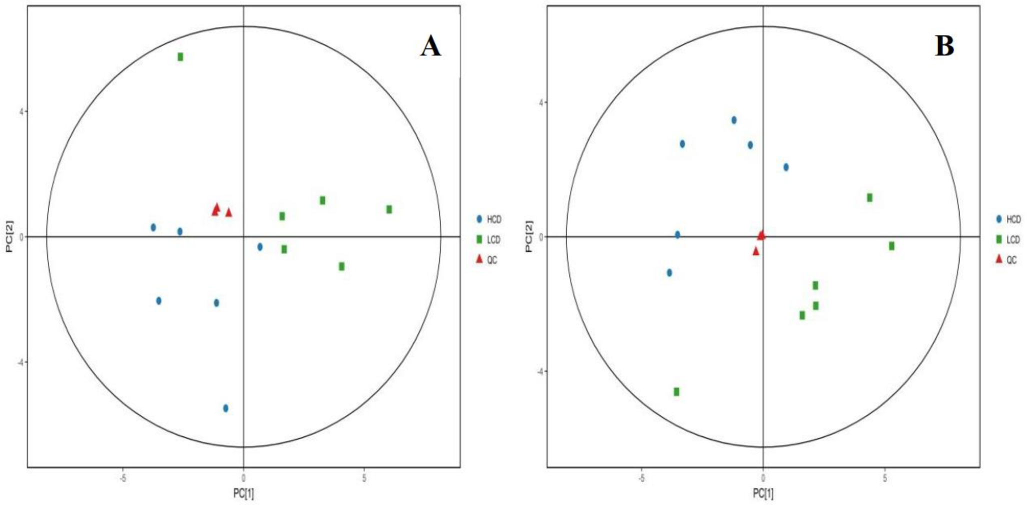

S3 Fig. Score scatter plot of the PCA model. A was derived from POS and B was derived from NEG. Red triangle were QC samples.
